# Protocol for a feasibility randomized trial of a social support intervention plus usual care versus usual care, targeting patients treated for cardiac disease who experience loneliness

**DOI:** 10.1186/s40814-023-01255-9

**Published:** 2023-02-06

**Authors:** Mitti Blakø, Anne Vinggaard Christensen, Ida Elisabeth Højskov, Pernille Palm, Selina Kikkenborg Berg

**Affiliations:** 1 Department of Cardiology, Rigshospitalet, Copenhagen University Hospital, Blegdamsvej 9, 2100 Copenhagen, Denmark; 2 Department of Heart- and Lung Surgery, RT, Rigshospitalet, Copenhagen University Hospital, Blegdamsvej 9, 2100 Copenhagen, Denmark; 3 Department of Cardiology B, Rigshospitalet, Copenhagen University Hospital, Blegdamsvej 9, 2100 Copenhagen, Denmark; 4 Faculty of Health and Medical Sciences, University of Copenhagen, Blegdamsvej 3B, N 2200 Copenhagen, Denmark

**Keywords:** Loneliness, Social support, Feasibility, Self-care, Cardiac rehabilitation

## Abstract

**Introduction:**

In patients treated for cardiac disease, loneliness is known to contribute negatively to health behavior, health outcome, and increase risk of cardiac and all-cause mortality. Even so, in health care research, social support interventional studies targeting patients who experience loneliness are lacking.

**Aim:**

To determine the feasibility of an individually structured social support intervention targeting patients treated for cardiac disease who experience loneliness.

**Design:**

A feasibility study based on randomized clinical trial design with 1:1 randomization to a 6-month social support program, plus usual care (intervention) versus usual care, (i.e., regular guidelines-based follow-up). Intervention: Patients classified as high risk lonely according to the High Risk Loneliness tool will be provided with an informal caregiver in the 6 months rehabilitation phase following cardiac disease treatment. The informal caregiver will be designated by the patient from the existing social network or a peer, depending on patients’ preferences. The core content of the intervention is through nurse consultations at baseline and 1, 3, and 6 months, to enhance and reinforce the informal caregiver’s competences to be a social support resource. The theoretical framework of the nurse consultations will be based on middle-range theory of self-care.

**Outcome:**

Feasibility will be evaluated in terms of acceptability and adherence according to predefined feasibility criteria. The preliminary effect of the intervention on patient-reported outcomes, health behaviors, and health outcomes will be evaluated in the intervention and the control group at baseline and 1, 3, 6, and 12 months.

**Discussion:**

The present study will contribute with knowledge on how to implement a feasible social support intervention targeting patients treated for cardiac disease who experience loneliness and, furthermore, investigate the preliminary effect on health behavior and health outcome in the early rehabilitation period.

**Trial registration:**

The trial is registered on clinicaltrials.gov (NCT05503810) 18.08.2022.

## Introduction

Cardiac disease is a major cause of impaired quality of life and the leading cause of mortality worldwide [1]. In Denmark, approximately 27,000 people are treated for cardiac disease, i.e., ischemic heart disease with coronary artery bypass grafting (CABG) or percutaneous coronary intervention (PCI), valve disease with transcatheter aortic valve replacement (TAVR) or surgical aortic valve replacement (SAVR), and arrhythmia with implantable cardioverter defibrillator (ICD), pacemaker implantation, or ablation [2]. One of the psychosocial factors that is known to contribute negatively to cardiac patients’ health behavior [3, 4], health outcome [5, 6], and cardiac and all-cause mortality [7–10] is loneliness. To illustrate, in a national study including 14,000 patients across cardiac diagnosis, it was found that feeling alone doubled 1-year mortality in analyses adjusted for baseline health status [9].

Loneliness can be defined as follows: “A distressing feeling that accompanies the perception that one’s social needs are not being met by the quantity or especially the quality of one’s social relationships” [11].

The link between loneliness and health outcomes is explained by two main hypotheses: (1) social support increases the feeling of trust and safety which helps to “buffer” the potentially harmful influences of stress-induced cardiovascular reactivity and (2) social support increases the motivation to make healthier choices [12, 13]. Besides, the impact of loneliness on health behaviors and health outcomes, and of similar importance, is that patients experience social support from network members as vital in order to handle the physical and psychological aftermath from in-hospital cardiac disease treatment [14–16]. This increased need for social support places patients with inadequate or sparse social support in a vulnerable situation in the early rehabilitation period.

Overall, the evidence on the link between loneliness and health has led to an elevated interest in intervening in this matter. Also, the European guidelines on cardiovascular disease prevention recommend the inclusion of a lack of social support as one of the core psychosocial risk factors in clinical practice [17]. However, in health care research, social support interventional studies targeting patients who experience loneliness is lacking. Therefore, it has yet to be demonstrated how to provide a feasible social support intervention and if improvement in perceived social support leads to improvements in health outcomes [18].

In the social integration literature [19–22], it is argued that interventions should be underpinned by a theoretical framework to increase the likelihood of achieving success. In this matter, social support interventions involving informal caregivers from patients existing social network have shown positive results. Also, caregiver-oriented strategies and psychological counseling have shown promising results on decreasing loneliness [23].

When structuring a social support intervention, evidence points that the intervention should be fitted to the needs and characteristics of the target population and, therefore, that it is advantageous to involve the target population in the design of the intervention [21]. In our own previous research, we have used patient involvement to illuminate potential preferences and barriers toward a social support intervention in patients with cardiac disease who experiences loneliness [24]. In this study, it was revealed that the intervention must meet the patient's individual preferences in relation to, e.g., the type of informal caregiver that provides the social support needed and intervention start-up time, frequency, and duration, in order to be an attractive proposition.

Based on findings from the patient involvement sessions, alongside the existing literature on loneliness and social support interventions, a study protocol for an intervention study targeting patients treated for cardiac disease who experience loneliness has been developed. As no previous studies have investigated an individually tailored social support intervention in this patient population, this initial study will be conducted as a feasibility trial [25]. Feasibility as a research method is chosen because it will provide valuable knowledge on acceptability, adherence, and attrition, as well as monitor potential deficiencies in the structure of the intervention.

## Study hypothesis

We hypothesize that an individually structured social support intervention targeting patients with cardiac disease who experience loneliness has the potential to decrease loneliness which in turn decreases stress-induced cardiovascular reactivity and promotes health behaviors which altogether will be reflected in patients’ wellbeing and in health outcomes.

## Study aims and objectives

The primary aim of the study is to determine the feasibility of an individually structured social support intervention targeting patients treated for cardiac disease who experience loneliness.

The secondary aim is to explore the preliminary evidence on the effect of the intervention on health behaviors and health outcomes. The results will be used to perform a sample size calculation in a potential future larger trial [26].

## Design and method

The protocol is based on reporting guidelines from the SPIRIT guideline for Standard Protocol Items for Clinical Trials [27] adapted as recommended when reporting protocols of feasibility trials [28].

## Trial design

A feasibility study based on randomized clinical trial (RCT) design. The RCT design with 1:1 randomization to a 6-month social support program, plus usual care (intervention) versus usual care, (i.e., regular guidelines-based follow-up [29], will provide preliminary evidence on the effect of the intervention on patient-reported outcomes, health behaviors, and health outcomes. To investigate the sustainability of a potential long-term effect of the intervention, the follow-up period will be 1 year.

### Sample size

**Fig. 1:**
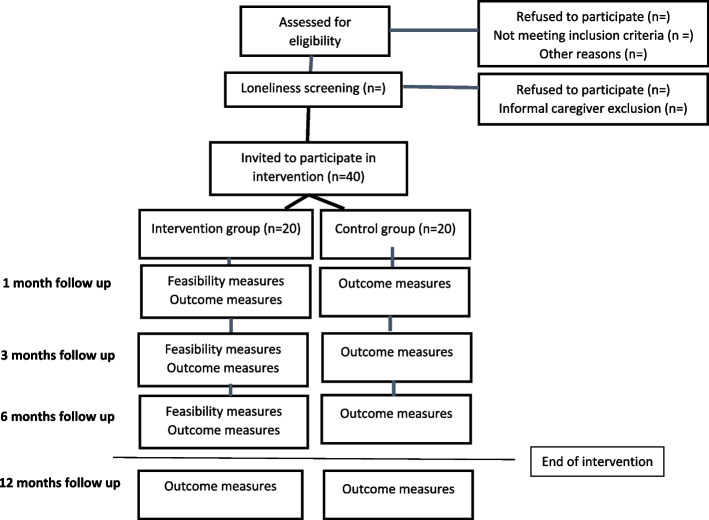
Flowchart

As the primary aim of this study is to test feasibility, there is no requirement for a formal power calculation [30]. Considerations about the sample size are related to the fact that no previous studies have investigated a social support intervention targeting patients with cardiac disease who experience loneliness and, therefore, no data exists on acceptability to the intervention. A sample size of 40 was chosen as it seems reasonable to exploratory investigate the feasibility, in particular acceptability, in a small sample and at the same time have a sample size big enough to investigate the additional aspects that are being assessed for feasibility [31]. Consequently, the study will include 20 patients and 20 informal caregivers in the intervention group and 20 patients in the control group. A flowchart of the trial is presented in Fig. 1.

## Trial population, eligibility criteria, and randomization

### Patients

Inclusion criteria: Three treatment groups, IHD (CABG or PCI), valve disease (TAVR, SAVR, surgical mitral valve procedures), and arrhythmia (ICD, pacemaker implantation or ablation) treated at Rigshospitalet and classified as high risk lonely according to the High Risk Loneliness (HiRL) tool [10], are given oral and written information about the study design and asked for permission to be contacted within 1 week from discharge to home. The HiRL screening tool has not yet been validated, but previous research suggest that the tool has a prognostic value for 1-year mortality, i.e., sensitivity 19.9% and specificity 89.5%, in patients treated for IHD. The HiRL screening tool is presented in Table 1. The final decision on participation must be taken by the patient within one week of discharge and patients will sign informed consent.

**Table 1:** High Risk Loneliness (HiRL) screening tool

Screening question	Answer	Point	Classification of high-risk loneliness
“Does it ever happen that you are alone even though you wish to be with others?”	No		≥ 1 point
	Yes, but rarely		
	Yes, sometimes		
	Yes, often	1	
“Do you have someone to talk to if you experience problems or need support?”	Yes, often		
	Yes, most of the time		
	Yes, sometimes		
	No, never or almost never	1	

Exclusion criteria: Patients who are unable to provide written consent; thus, patients with severe cognitive or physical dysfunction will not be approached.

Randomization: Patients will be identified and screened consecutively. Randomization is completed using the web-based tool Randomizer for Clinical Trials. Patients will be stratified according to gender.

Patients who accept participation and who are included in the intervention group will receive further oral and written information about the intervention structure and sign informed content. In relation to this, they will be asked about their preference for the choice of an informal caregiver and for the structure of the intervention.

### Informal caregivers

The informal caregiver is identified by the patient from two of the following options:

Social network member: This type of informal caregiver will be designated by the patient from the existing social network (e.g., a partner, friend, or neighbor), and the patient will be asked to initiate the contact with the social network member. If he/she accepts to be an informal caregiver, the intervention staff will contact the informal caregiver and give oral and written information, and if acceptance is confirmed, an inclusion-consultation will be performed subsequently. The consultation can be performed in-person or remote (phone or virtually). This consultation aims at investigating the potential caregiver’s prerequisites, resources, and motivation to participate with minimal risk of suffering from caregiver burden.

Exclusion criteria: Potential informal caregivers who are assessed as not having the necessary prerequisites or do not speak or understand Danish.

If the social network member does not accept to be an informal caregiver or if he/she is deemed to be at risk of caregiver burden, the patient will be given the opportunity to point out an alternative social network member to be an informal caregiver or to choose a peer as an informal caregiver.

Peer: This type of informal caregiver is defined as a person with similar disease as the recipient. The peer will be recruited by the Danish Heart Foundation from among volunteers in the existing peer support program.

## Overall intervention structure

The core content of the intervention is through nurse consultations to enhance and reinforce the informal caregiver’s competences to be a social support resource. The intervention structure is based on the evidence on the positive impact of support provided by a peer (a person with similar disease as the recipient) [18, 32] or by a network member [23, 33] and the findings from patient involvement interview sessions performed by the project group [24]. Therefore, the patient will be provided with an informal caregiver in the long-term (6 months) rehabilitation phase following cardiac disease treatment. The role of the informal caregiver as a social support resource will be the same independent of which type of caregiver (social network member or peer) the patient has chosen. After choosing the type of informal caregiver, the patient will be asked about preferred frequency and form of contact with the informal caregiver. The patient and the informal caregiver are encouraged to be in contact a minimum of once a week in-person or remote (phone or virtually). The intervention staff will contact the informal caregiver and ask for acceptance of the suggested structure. If a need for adjustment of frequency or form occurs in the intervention period, patients and informal caregivers may arrange this together or in consultation with the intervention staff.

Furthermore, the patients will receive motivational text messages intended to enhance the supportive environment [34]. The text messages will be formulated by the research group and will be sent automatically to the patient on Mondays between 1 and 3 pm by a text message gateway “cpsms.” The wording of the text messages can, for example, be “*The choices you make today shape the life you live tomorrow*” or “*Success is the sum of small efforts repeated*.”

At enrollment, 1 month, and 3 and 6 months, an intervention nurse will contact the informal caregiver and provide guidance and counseling based on a theoretical framework derived from complementary fields, i.e., psychology with the theory of dyadic processes [12, 35] and nursing with self-care theory [36, 37]. These theoretical frames will inform the intervention nurse on which domains to focus on when guiding the informal caregiver. An intervention guide for the nurse is used in all nurse consultations. A description of the aim and method for each consultation described in the intervention guide is shown in Table 2.

**Table 2:** Aim and method for each nurse consultation with the informal caregiver

Enrollment	One month	Three months	Six months
• For the nurse, to gain insight into the informal caregiver's prerequisites • To provide information to the informal caregiver about the role as “HeartBuddy” • Guidance and counseling in emotional support, i.e., encourage the informal caregiver to ask the patient open and curious questions about their well-being and everyday life, and, to listen without prejudice or neglecting patient thoughts and feelings • Guidance and counseling in informational support related to patients’ health behavior and self-care, i.e., maintenance, monitoring, and management	• Gain insight into the relational processes between patient and informal caregiver and provide guidance in relation to potential challenges • Guidance and counseling in emotional support • Guidance and counseling in informational support related to patients’ health behavior and self-care	• Gain insight into the relational processes between patient and informal caregiver and provide guidance in relation to potential challenges • Guidance and counseling in emotional support • Guidance and counseling in informational support related to patients’ health behavior and self-care	• Guidance in relation to ending the role as “HeartBuddy”

The central themes are furthermore described in an easy-to-read langue, to be handed out to the informal caregiver as a pamphlet “Guide to HeartBuddy.”

Both patient and informal caregiver will be provided with the possibility of contacting an intervention nurse through an open hotline (workdays, daytime) if any additional questions arise during the intervention period.

## Outcomes

### Feasibility

The outcomes of the feasibility study will inform the design of a potential upcoming randomized control trial (RCT). The feasibility will be evaluated in terms of acceptability and adherence [38, 39]. Feasibility criteria are based on the balance between the financial and logistical resources of the intervention and the expected effect.

The feasibility criterion for acceptability in patients is supported if 25% of patients who are screened as lonely agree to participate in the trial.

The feasibility criteria for acceptability in social network members is supported if 50% of invited social network members accept participation and are assessed to have the prerequisites to be an informal caregiver.

The feasibility criteria for adherence in patients is supported if 75% adhere to the intervention, i.e., have contact with the informal caregiver once a week for a minimum of 8 out of 12 weeks.

The feasibility criteria for adherence in informal caregivers (social network member or peer) is supported if 75% participate in three out of four consultations. An overview of the feasibility criteria is presented in Table 3.

**Table 3:** Feasibility measurement, measurement definition, and feasibility criteria

Feasibility measurement	Measurement definition	Feasibility criteria
Acceptability	• Percentage of eligible patients who agree to participate in the trial • Patients’ choice of type of informal caregiver *Measures of social network members as informal caregivers*: • Percentage of potential caregivers who accepts to be an informal caregiver • Percentage assessed to have prerequisites to be an informal caregiver	25% of patients who are screened and found to be lonely agree to participate in the trial 50% of invited social network members accept participation and is assessed to have prerequisites to be an informal caregiver
Adherence	• Percentage of informal caregivers participating in all three intervention consultations with the trial staff • Percentage in contact with the patient at least once a week face to face, by phone or virtually	75% of informal caregivers participate in three out of four consultations 75% of included patients adhere to the intervention, i.e., having contact with the informal caregiver once a week for a minimum of 8 out of 12 weeks
Loss to follow-up	• Percentage of patients and informal caregivers who do not complete the trial	A maximum of 25% of informal caregivers do not attend two out of three intervention consultations with the trial staff attend A maximum of 25% of patients do not complete the trial
Resource consumption	• Time resources used to include study participants • Time resources used to complete the three nurse consultations	N/A for feasibility

In feasibility and pilot testing, it is furthermore recommended that the resources used to complete the study is monitored [30]. This will be done by keeping a record of time resources used to include study participants and to complete the four nurse consultations (enrollment, 1 month, 3 and 6 months) and also frequency and time used on the additional questions from patients and informal caregivers to the open hotline.

### Health behaviors and health outcomes

All outcomes will be measured in the intervention and control groups at baseline and 1, 3, 6, and 12 months. Demographic characteristics (age, gender) and treatment group (IHD, valve disease or arrhythmia) will be obtained from medical record.

**Fig. 2:**
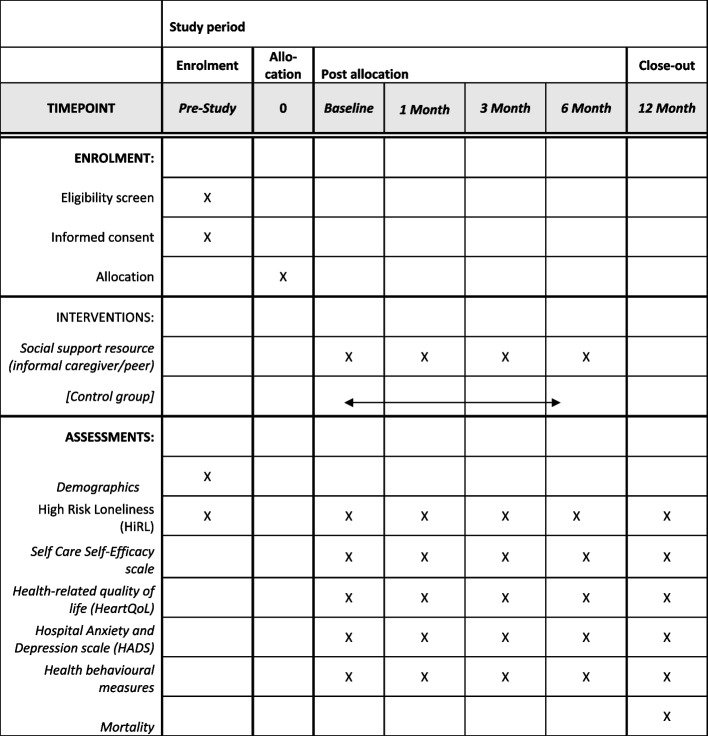
Schedule of enrollment, interventions, and data collection

Other outcomes will be obtained patient reported in REDCap. The primary outcome of interest, loneliness, is measured with HiRL tool [10]. The secondary outcome of interest is measured with the following questionnaires: Self-care: Self Care Self-Efficacy scale (Cronbach’s alpha 0.93) [40], health-related quality of life (HeartQoL) (Cronbach's alpha ≥ 0.80) [41], and Hospital Anxiety and Depression scale (HADS) [42] (in cardiac patients: Cronbach’s alpha 0.87 for HADS-A and 0.82 for HADS-D) [43]. Measures on health behaviors, i.e., smoking, alcohol consumption, weight, physical activity, and participation in cardiac rehabilitation, will be patient reported. An overview of outcome measurements and related measurement tools is presented in Fig. 2.

## Data analysis

As recommended for the analysis of pilot studies [44], descriptive statistics will be presented as mean and standard deviations (SD) for continuous variables and frequencies and percentages for categorical variables at baseline and follow-ups (1, 3, 6, and 12 months).

To examine the comparability of the intervention group and the control group, baseline characteristics of the groups will be compared using Pearson’s chi-square or Fisher’s exact test for categorical variables and a *t*-test or Mann–Whitney test for continuous variables. Similar descriptive comparison of health outcome variables by group will be performed at follow-ups. The primary analyses will be performed according to the intention-to-treat principle.

To improve statistical efficiency, the primary outcome, i.e., loneliness, will be analyzed with a regression model adjusted for the stratification variables and the baseline variables, as recommended by the American Medical Association [45] and the European Medicines Agency [46].

Note, we do not aim to investigate statistically significant differences between groups as this is a feasibility trial. Therefore, no power calculation will be conducted. Rather, the measurement of health behaviors and health outcomes aims to give an indication of the potential variability in the outcome measures, which will be used to inform the power calculation for a future RCT.

A lost to follow-up analysis will be conducted for gender, age (higher or lower than mean), treatment procedure, and type of informal caregiver.

All statistical analyses will be performed using SAS Enterprise 7.1.

## Discussion

A social support intervention targeting patients treated for cardiac disease who experience loneliness has the potential to decrease the negative impact on health from cardiovascular reactivity and to promote condition management in the early rehabilitation period. Consequently, a social support intervention may diminish the inequality in health behavior and health outcomes we see in patients who experience loneliness. To date, the type of support, with the intensity and the duration of the intervention needed to bring about changes in the experience of loneliness and the process that mediates health behavior and health outcomes, is scarce. The present study will contribute with new knowledge on how to implement a feasible social support intervention and, furthermore, investigate the preliminary evidence on the effect on health behaviors and health outcomes. The added knowledge will inform the design of a potential upcoming RCT.

### Trial registration

The trial is registered on ClinicalTrials.gov (NCT05503810) 18.08.2022.

Positive, negative, and neutral results will be published in anonymized form.

## Data Availability

Data material can be requested upon reasonable request.
